# Deregulation of the phosphatase, PP2A is a common event in breast cancer, predicting sensitivity to FTY720

**DOI:** 10.1186/1878-5085-5-3

**Published:** 2014-01-25

**Authors:** Shawn Baldacchino, Christian Saliba, Vanessa Petroni, Anthony G Fenech, Nigel Borg, Godfrey Grech

**Affiliations:** 1 Department of Pathology, Medical School, University of Malta, Msida MSD2090, Malta; 2 Department of Clinical Pharmacology and Therapeutics, University of Malta, Msida MSD2090, Malta

**Keywords:** Predictive biomarkers, PP2A, Therapeutic groups, Personalised medicine

## Abstract

**Background:**

The most commonly used biomarkers to predict the response of breast cancer patients to therapy are the oestrogen receptor (ER), progesterone receptor (PgR), and human epidermal growth factor receptor 2 (HER2). Patients positive for these biomarkers are eligible for specific therapies such as endocrine treatment in the event of ER and PgR positivity, and the monoclonal antibody, trastuzumab, in the case of HER2-positive patients. Patients who are negative for these three biomarkers, the so-called triple negatives, however, derive little benefit from such therapies and are associated with a worse prognosis. Deregulation of the protein serine/threonine phosphatase type 2A (PP2A) and its regulatory subunits is a common event in breast cancer, providing a possible target for therapy.

**Methods:**

The data portal, cBioPortal for Cancer Genomics was used to investigate the incidence of conditions that are associated with low phosphatase activity. Four (4) adherent human breast cancer cell lines, MDA-MB-468, MDA-MB-436, Hs578T and BT-20 were cultured to assess their viability when exposed to various dosages of rapamycin or FTY720. In addition, RNA was extracted and cDNA was synthesised to amplify the coding sequence of PPP2CA. Amplification was followed by high-resolution melting to identify variations.

**Results and conclusion:**

The sequence of PPP2CA was found to be conserved across a diverse panel of solid tumour and haematological cell lines, suggesting that low expression of PPP2CA and differential binding of inhibitory PPP2CA regulators are the main mechanisms of PP2A deregulation. Interestingly, the cBioPortal for Cancer Genomics shows that PP2A is deregulated in 59.6% of basal breast tumours. Viability assays performed to determine the sensitivity of a panel of breast cancer cell lines to FTY720, a PP2A activator, indicated that cell lines associated with ER loss are sensitive to lower doses of FTY720. The subset of patients with suppressed PP2A activity is potentially eligible for treatment using therapies which target the PI3K/AKT/mTOR pathway, such as phosphatase activators.

## Overview

Breast cancer accounts for approximately 23% of cancer cases in females and is responsible for 14% of cancer-related deaths in females [1]. The classification of breast cancer is shifting to an expression-oriented classification. Histological assessment is still a vital tool for assessing prognosis through stage (lymph node status, tumour size and grade). Immunohistological investigations are instrumental in defining subtypes and for planning treatment strategies. Breast cancer is being classified into four broad categories: luminal A, luminal B, the human epidermal growth factor receptor 2 (HER2)-enriched and basal-like. The luminal class indicates oestrogen receptor (ER) and/or progesterone receptor (PgR) positivity but a negative HER2 and a low Ki-67 expression (<14%) for the A subtype. Luminal B is subdivided into the HER2 negative along with high Ki-67 and the HER2 positive. The HER2-enriched are negative for both the ER and PgR while the basal-like class is in its majority negative for all threehormone receptors (triple negative). ER and PgR receptor expression confers susceptibility to endocrine therapy whereas HER2-positive breast cancer is treated with trastuzumab (anti-HER2 treatment) [2,3].

Treatment of the basal-like class is currently based on cytotoxic drugs and have a worse overall and disease-free survival [2,4]. A small subclass, referred to as the special histological types (being medullary and adenoid cystic carcinomas) can still be eligible to endocrine treatment [2]. Interestingly, the basal subtype shows a higher occurrence of phosphatidylinositol 3-kinase (PI3K) pathway activation [5].

Receptor tyrosine kinases such as HER2 and insulin-like growth factor 1 (IGF-1) receptor activate the PI3K pathway, initiating a cascade of signals. Active PI3K generates phosphatidylinositol 3,4,5 triphosphate (PIP3), which serves as an anchor for Pleckstrin homology (PH)domain-containing proteins, including both adaptor molecules such as GRB2-associated binding protein 2 (Gab2) and docking protein 1 (Dok1) and kinases such as tec protein tyrosine kinase (Tec), Bruton agammaglobulinemia tyrosine kinase (Btk), phosphoinositide-dependent kinase-1 (PDK1) and v-akt murine thymoma viral oncogene homolog kinase (AKT) [6-9]. Activation of AKT increases cell cycle progression and maintains mammalian target of rapamycin (mTOR) signalling resulting in enhanced cell proliferation and survival, respectively. The PI3K pathway is attenuated by phosphatases, including phosphatase and tensin homolog (PTEN) which dephosphorylates PIP3 [10] and protein serine/threonine phosphatase type 2A (PP2A) which inactivates mTOR effectors [11].

PP2A is a complex of multiple subunits that interact to define the enzyme’s substrate targets. Moreover, other regulatory subunits may bind to the complex to regulate its activity. Hence, understanding the structure of PP2A and its interactions with associated proteins sheds light on its regulation and its substrates. PP2A controls the phosphorylation of a number of kinases involved in cell signalling by catalysing dephosphorylation of the downstream intracellular proteins. As PP2A activity regulates various pathways, its downregulation may be involved in the progression of multiple tumour types [12,13]. The role of PP2A has been studied extensively in various cellular models, and deregulation of PP2A and its subunits (alpha4 and SET) are implicated in enhanced proliferation and differentiation block in haematopoietic progenitor cultures [14]. Missense mutations in the structural subunits, PPP2R1A and PPP2R1B, result in suppressed PP2A complex formation. Loss of heterozygosity is observed in a significant percentage of breast, lung, ovarian, colon and liver and melanoma malignancies and to a lesser extent in non-Hodgkin’s lymphomas and chronic lymphocytic leukaemia [15-20]. Somatic missense mutations were also detected in PPP2R1A in high-grade serous endometrial tumours [21]. A particular study has shown that PPP2R1B is mutated in 13% of breast cancers, and these mutations result in defective attachment of the B and C subunits. Whole gene deletions, missense, frame-shift, internal and C-terminal protein deletions are likely to hinder the formation of the PP2A active core dimer. Half of the breast cancers investigated had a low expression of PPP2R1B, contributing to malignant transformation [17,22]. In fact, PPP2R1B has been described as a tumour suppressor gene as it is often found to be deleted in human breast cancer. In addition, inactivation of PP2A by phosphorylation at tyrosine 307 is significantly correlated with HER2-positive tumour progression [23].

Loss of function of the phosphatases PTEN [24] and inositol polyphosphate 4-phosphatase type II (INPP4B) [25] is associated with aggressive basal-like breast carcinoma. PTEN, INPP4B and PP2A are known antagonists of AKT phosphorylation; hence, loss of phosphatase function leads to increased AKT activation. Interestingly, BRCA1 is known to activate PP2A, a phosphatase that dephosphorylates AKT at threonine 308 (T308) and serine 473 (S473) [26,27]. This is supported by the findings that loss of BRCA1 activity leads to increased AKT activity [28] and reduced PP2A activity [26]. In addition, BRCA1 is known to bind phosphorylated AKT (p-AKT) and lead to its ubiquitination [29]. In fact, an enhanced stability and higher expression of p-AKT can be found in BRCA1 mutants, in which the mutant BRCA1 lacks the ability to bind to p-AKT [28].

Overexpression of the PP2A biomarkers p-S6K and p-AKT has been described in breast and ovarian tumours potentially reflecting attenuated PP2A activity [30-33]. New insights into the mechanism of PP2A regulation in solid tumours form the basis of potential identification of variants that affect the phosphatase activity. The regulating subunits CIP2A (cancerous inhibitor of PP2A, also known as KIAA1524) and SET bind to the PP2A complex and specify its targets. These subunits were found to be overexpressed in various tumours including breast, colon and renal tumours, respectively [34-36].

In this study, we scanned for mutations in the PP2A catalytic subunit, PPP2CA transcript (mRNA) in various breast cancer cell lines. Publically available datasets (cBioPortal) were used to investigate the frequency of mutations and expression of the PP2A complex components and regulatory subunits. Of interest, the cBioPortal for Cancer Genomics shows that the PP2A complex is deregulated in 59.6% of basal breast tumours. Investigations to determine the sensitivity of a panel of breast cancer cell lines to FTY720, a PP2A activator, indicated that cell lines associated with ER loss are sensitive to lower doses of FTY720. Interestingly, using the specific inhibitor of the mTOR kinase, rapamycin, on the same panel of breast cancer cell lines resulted in a different sensitivity profile. Our interest in the use of FTY720 originates from the observations in our preliminary studies showing enhanced sensitivity of a BRCA1 mutant breast cancer cell line to FTY720 (unpublished).

These cases are eligible to pharmaceutical inhibition of the PI3K pathway and potentially activation of the phosphatase PP2A. Activation of PP2A will allow not only targeting of the deregulated PI3K pathway, including kinase mutants and cells with a low PTEN expression, but also BRCA1 mutants due to the sensitivity conferred by the lower PP2A activity.

## Methods

### Data mining using cBioPortal for Cancer Genomics

A data portal (cBioPortal for Cancer Genomics [37]), available at http://www.cbioportal.org was used to measure the incidence of conditions that are associated with low phosphatase activity, as per the criteria in Table 1[37]. The database query was based on deregulation (mutant and altered expression) of the PP2A complex components and upregulation (expression) of the inhibitory regulators of the complex.

**Table 1:** Criteria used to select breast cancer cases with deregulated PP2A in the cBioPortal for Cancer Genomics

**Variable**	**Criteria**
Selects low expression (including deletions) of one of the pp2a complex components	
PPP2CA	Homozygous deletions; downregulation (fold change < −2)
PPP2CB	
PPP2R2A	
Selects high expression, including amplification, of the inhibitory regulatory subunits	
KIAA1524 (cip2a)	Amplification; upregulation (fold change >2)
SETBP1	
SET	
IGBP1 (alpha4)	

### Cell lines used and culturing conditions

Four (4) adherent human breast cancer cell lines were used in the study, namely MDA-MB-468, MDA-MB-436, Hs578T and BT-20. The cells were cultured in sterile T-25 flasks in an incubator at 37°C, having an atmosphere of 5% CO_2_ and 98% humidity, using RPMI 1640 medium containing 10% foetal bovine serum (FBS) and 1% penicillin/streptomycin. Passaging was carried out when the cells reached around 90% confluence.

### RNA extraction and cDNA synthesis

Cell pellets were prepared from 3 to 5 × 10^6^ cells. Cells were lysed in QIAzol and stored at −80°C. RNA was extracted using RNeasy isolation kit (Qiagen, Venlo, The Netherlands). Quality was validated by spectrophotometry 260/280 and 260/230 ratios using the nanodrop, and the integrity was checked using the Agilent Bioanalyser, Santa Clara, CA, USA.

RNA was reverse transcribed into cDNA using the Quantitect Reverse Transcription Kit from Qiagen. This kit provides high cDNA yields even from low abundance transcripts and eliminates genomic DNA contamination effectively.

### High-resolution melting

Primers were designed to amplify the coding sequence of PPP2CA into individual 250 to 300 bp overlapping fragments by polymerase chain reaction (PCR). Amplification was followed by HRM, using a Qiagen Rotor-Gene instrument. Variants were characterised by their melting temperatures through the distinct kinetics of fluorescence loss during HRM. Shifts in the melting temperature or HRM peaks were identified visually using the Rotor-Gene software and also using the Rotor-Gene ScreenClust HRM Software. The latter software processes data generated throughout the amplification and HRM in order to segregate samples into potentially distinct groups. To characterise the variations, the whole PPP2CA coding sequence was amplified using the terminal primers in a conventional PCR. Products were purified using the Wizard® SV Gel and PCR Clean-Up System (Promega, Madison, WI, USA). The full-length PPP2CA amplicons were sequenced to confirm the HRM results.

### Cell line sensitivity assays

The adherent human breast cancer cell lines described above were plated in 24 well plates to determine two seeding densities to be used for the sensitivity assays that run for 48 h. Selection of the cell densities was based on (1) reaching a confluence of 80% at the time point of drug administration and in different wells (2) reaching a confluence of 80% following 72 h of seeding. Cells were plate in triplicates and incubated for 24 h to allow adherence. At 24 h, FTY720 was added at a final concentration ranging from 0.5 to 25 μM, while rapamycin was added in a separate experiment at a final concentration ranging from 10 to 100 μM. A vehicle control and an untreated control were added. An MTT (3-(4,5-dimethylthiazol-2-yl)-2,5-diphenyltetrazolium bromide) assay was performed at the end of the incubation periods (24 and 48 h) to determine cell viability. A replicate experiment to confirm the results was done.

## Results

In this study, no mutations were identified in the PPP2CA coding sequence in various breast cancer cell lines. *In silico* analysis using a publically available dataset, the cBioPortal for Cancer Genomics, shows that the protein phosphatase 2 (PP2A) complex is deregulated in 59.6% of basal breast tumours. In a panel of breast cancer cell lines, ER loss correlates with sensitivity to lower doses of FTY720.

### PPP2CA coding sequence is highly conserved

Screening of PPP2CA coding sequence for mutations, using HRM analysis, could not detect mutations in the adherent human breast cancer cell lines and in an additional panel of 25 tumour cell lines (14 haematological and 11 solid tumour cell lines). The wild-type sequence was confirmed in the human breast cancer cell lines used in this study (data not shown) by re-sequencing.

### Protein phosphatase type 2A (PP2A) is deregulated in 59.6% of basal breast tumours

**Figure 1:**
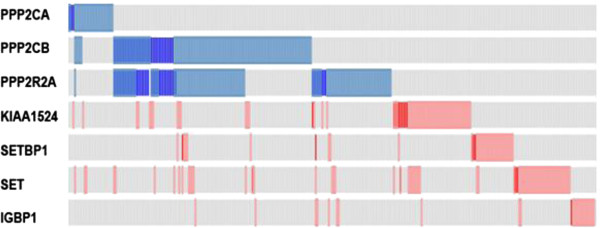
**Distribution of aberrations associated with low PP2A activity.** In this analysis, all subtypes of breast cancer patients were included. Blue bars indicate low expression (light) or homozygous deletion (dark), while the red bars indicate high expression (light) or amplification (dark)(KIAA1524 is equivalent to CIP2A; IGBP1 is equivalent to alpha4).

Analysing the results from datasets originating from RNAseq in the cBioPortal, and using the criteria described above, 46.7% (245 cases out of 525 eligible cases) of all the subtypes of breast cancer patients either had a low expression, including deletions, of one of the PP2A complex components or a high expression, including amplification, of the inhibitory regulatory subunits (Figure 1). Interestingly, the criteria were generally mutually exclusive, except for PPP2CB and the PPP2R2A which can occur simultaneously.

Surprisingly, 8.6% of the patients either had a high expression of CIP2A (KIAA1524) or a high expression of SET, implying that the PP2A complex is sequestered from the negative feedback on mTOR signalling. Although overall, the PPP2CA expression is low in 4.2% of the patients, the low expression was associated mainly with the basal phenotype. In addition, PP2A deregulation is associated with 59.6% of the basal phenotype (Table 2).

**Table 2:** Incidence distribution of aberrations associated with low PP2A activity in the various breast cancer subtypes

	**Basal (*N*** **= 99)**	**Luminal A (*N*** **= 235)**	**Luminal B (*N*** **= 133)**	**HER2-enriched (*N*** **= 58)**	**Total (*N*** **= 525)**
PPP2CA	17.2%	0.4%	2.3%	1.7%	4.2%
PPP2CB	21.2%	14.9%	30.8%	25.9%	21.3%
PPP2R2A	18.2%	11.9%	24.1%	24.1%	17.5%
CIP2A	18.2%	3.0%	10.5%	10.3%	8.6%
SETBP1	4.0%	6.4%	7.5%	0.0%	5.5%
SET	15.2%	2.6%	8.3%	24.1%	8.8%
IGBP1	4.0%	7.7%	2.3%	3.4%	5.0%
Total PP2A deregulation	59.6%	35.3%	51.9%	58.6%	46.7%

### Cell lines associated with ER loss are sensitive to lower doses of FTY720

**Figure 2:**
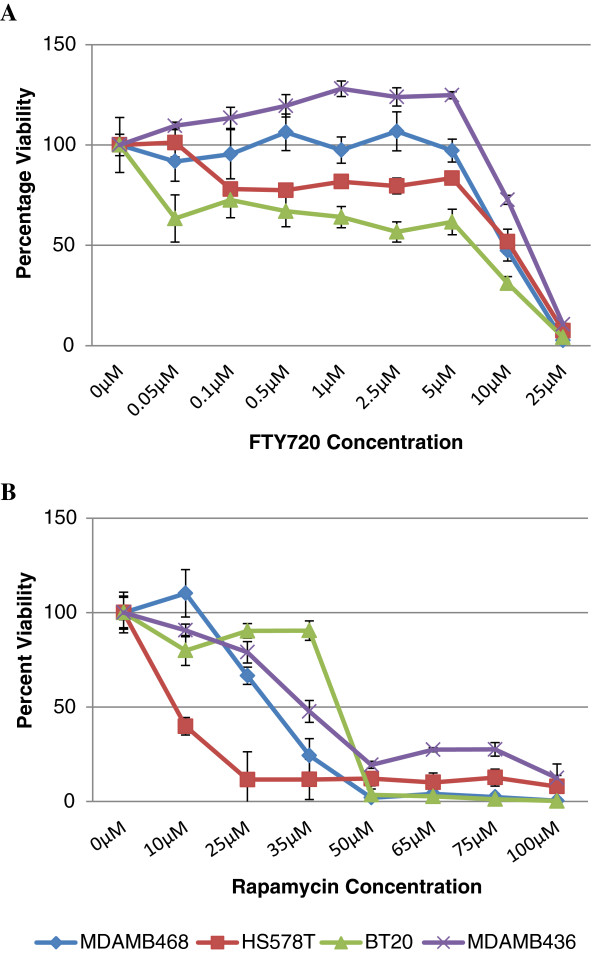
**Viability assays of breast cancer cell lines.** Cells were cultured in the presence or absence of FTY720 **(A)** or rapamycin **(B)** using a concentration gradient and absorbance was measured after 48h of drug exposure. Each data point is an average of three independent experiments, with triple measurements for each experiment. The viability of each well is expressed as a percentage normalised to vehicle control cells. The standard error is indicated using error bars. The blue lines plot the percent viability of MDAMB468, the red line Hs578T, the green line BT20 and the purple line of MDAMB436.

The cell lines, BT20 and Hs578T, were found to be sensitive to 0.05 and 0.1 μM FTY720, respectively. A significant reduction in the metabolism of these cells persisted up to 5 μM, with a percentage viability of 60% in BT20 and 80% in Hs578T, when compared to the vehicle control culture (Figure 2A). Of interest, both these cell lines have no ER expression or express a truncated, inactive ER. In contrast, the BT20 cell line is resistant to rapamycin, while Hs578T was extremely sensitive to low doses with an IC_50_ reached at 10 μM (Figure 2B). The other breast cancer cell lines studied proliferated in the presence of 5 μM FTY720 and reached an IC_50_ at approximately 35 μM of rapamycin.

### Results interpretation

In this study, we observed that the PPP2CA coding sequence is not polymorphic in various tumour cell lines tested. The PPP2CA gene sequence is highly conserved throughout species (ENSEMBLE version release 72 [38]). Currently, 25 missense variants, 4 stop codon gains and 2 frame-shift variants have been described in literature. Although the mutational analysis did not yield any missense mutations, *in silico* analysis of publically available RNAseq datasets (cBioPortal) indicated clearly that deregulation of the PP2A complex mainly occurs through altered expression of the subunits and inhibitory regulators. Indeed, 17.2% of cases with the basal subtype showed low expression or homozygous deletion of the catalytic subunit, PPP2CA; 18.2% were associated with high expression of CIP2A and 15.2% with high expression of SET (Table 2). Overall, PP2A is deregulated in 59.6% of basal breast tumours.

Cytoplasmic CIP2A overexpression at mRNA and protein levels correlates with high tumour grade and aggressiveness in breast cancer patients [34,39]. Overexpression of CIP2A in normal tissues is only restricted to brain, prostate and testis, but it is undetectable in normal breast tissue. Overexpression is a common occurrence in malignancy including colon, prostate, ovarian cancer and head and neck squamous cell carcinomas and is most often related to the more aggressive cases of high-grade or advanced tumour stages [35,40-43]. CIP2A overexpression clustered mainly with basal-like breast tumours. Out of 40 breast cancer cell lines, basal-like breast tumour cell lines exhibited the highest CIP2A overexpression [44]. Similarly, SET and alpha4 (IGBP1) are implicated in promoting the progression of disease and enhanced proliferative signals in leukaemogenesis [14,45]. Of interest, expression of the alpha4 inhibitory subunit is dependent on the efficiency of translation initiation, promoted by the mTOR pathway. Hence, expression should be measured at a protein level, and the incidence of high alpha4 expression is expected to be greater than 3% (Figure 1, IGBP1). Although no data on the protein expression of alpha4 is available in the cBioPortal, the Human Protein Atlas hints to an increased expression of alpha4 protein in breast tumour as opposed to normal breast tissue through immunohistochemical analysis.

**Figure 3:**
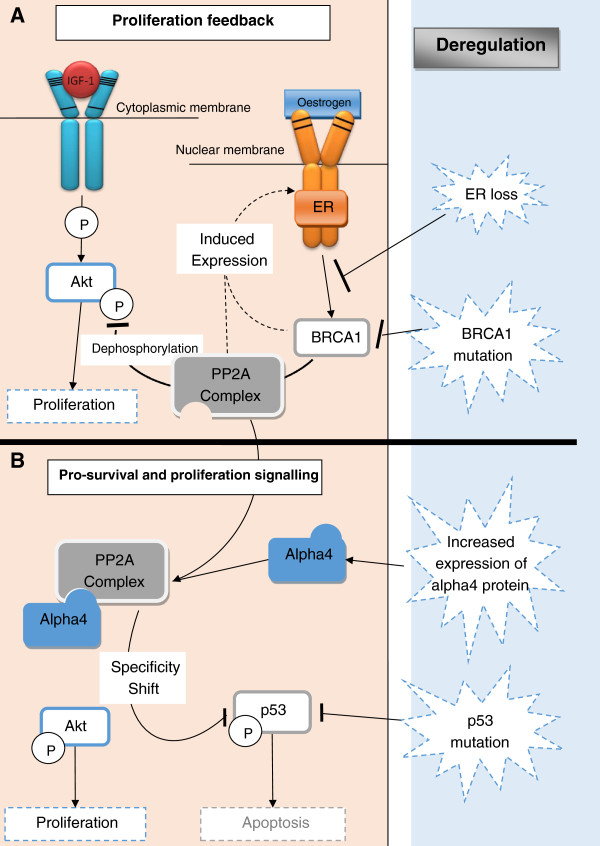
**Schematic diagram illustrating the dual role of PP2A complex.** PP2A complex attenuates the proliferative signals transduced via the AKT/mTOR pathway. Activation of PP2A is dependent on ER-induced BRCA1 expression. Attenuation mechanism following **(A)** haploinsufficiency or loss of function of ER or BRCA1 or **(B)** increased expression of the inhibitory regulatory subunit, alpha4. Alpha4 sequestrated the PP2A complex, maintaining AKT/mTOR signals and shifts the specificity of the PP2A-alpha4 complex towards pro-survival signals, via dephosphorylation of p53.

Binding of alpha4 to the PP2A complex shifts the phosphatase activity from a negative feedback mechanism that attenuates proliferation (mTOR signalling) to a pro-survival activity (Figure 3) through inactivation of p53 [46-48]. To support the importance of substrate specificity following regulatory subunit binding to the PP2A complex, studies indicate that the SET-PP2A complex activates the ERK/MAPK pathway inhibiting apoptosis [49] and the CIP2A-PP2A complex releases inhibition on p-AKT and c-Myc and hence promotes pro-proliferative signals [50]. This implies that the PP2A complex shifts from a tumour suppressor to a promoter of oncogenic signals.

Deregulation of p53 is a frequent event in breast cancer associated with 33% of breast cancer patients [37]. Formation of the PP2A-alpha4 complex is an alternative mechanism promoting cell survival through p53 inactivation (Figure 3).

Another hallmark of the molecular basis of breast cancer is the loss of function of BRCA1. BRCA1 mutations occur in approximately 50% of hereditary breast cancers, but a low expression of this gene was observed in 40% to 80% of cases [51]. Interestingly, BRCA1 has been shown to activate PP2A [26], and a low BRCA1 expression correlate with elevated phosphorylation of AKT [51]. In addition, IGF-1-induced activation of p-AKT is inhibited by FTY720 in the BRCA1 mutant cell line, HCC1937 (data not shown), indicating that loss of function of BRCA1 leads to low phosphatase activity and higher sensitivity to PP2A activators.

In this study, viability assays showed that cell lines associated with oestrogen receptor (ER) loss are sensitive to lower doses of FTY720. ER-negative breast cancer cell lines have a suppressed PP2A activity, when compared to breast cancer cell lines expressing ER receptors [52], supporting the higher sensitivity to the phosphatase activator, FTY720. ER-dependent BRCA1 expression [53] provides a plausible mechanism (Figure 3), since low expression of BRCA1 in ER-negative cell lines results in a reduced ability to activate PP2A. Of interest, viability of the ER-negative cell line, BT-20, was unaffected by rapamycin up to a high dose (35 μM). Hence, the sensitivity to PP2A activation in the BT-20 cell line is not dependent on the attenuation of the mTOR kinase, which is specifically inhibited by rapamycin. This suggests that the formation of the core PP2A complex, following pharmacological activation by FTY720, dephosphorylates the mTOR downstream effectors, 4EBP and S6K, and concurrently releases the block on the p53 pathway. Our results suggest that markers can be used to predict sensitivity to FTY720 and that the pharmacological activation of PP2A is an attractive therapeutic modality that simultaneously targets proliferative signals and releases PP2A-dependent p53 inhibition. In addition, the use of FTY720 is a potential alternative therapy to inhibitors of the kinase mTOR, which proved to have limited success due to resistance to therapy. A panel of biomarkers that predict sensitivity to mTOR inhibitors [54] and activation of the phosphatase, PP2A, merit further investigation to allow characterisation of the potential therapeutic group. This approach utilise the use of efficacy biomarkers, assessing the beneficial effects of a clinically available treatment, promoting personalised medicine [55].

### Expert recommendations

The characterisation of the molecular mechanism of disease allows classification of patients into subtypes and potentially identifies specific targets for therapeutic intervention. Tyrosine kinase mutations are central to specific targeted therapy. Investigation of kinase deregulation within particular patient groups has led to identification of mutant tyrosine kinases associated with disease progression and therapy modulation. Biomarker-specific therapies emerged, taking a leading role in guidedtherapy. Loss of function mutations in the phosphatase, PP2A, and/or an enhanced PP2A inhibition due to increased expression of PP2A regulators (such as CIP2A, SETBP1) will provide a new classification of patients in various malignancies. Of interest, these subtypes will provide the basis to investigate the use of PP2A activators as therapeutic drugs, hence promoting personalised medicine. Current diagnostic panels assessing risk, early diagnosis and patient management require a more robust platform, integrating classifier biomarkers, clinical data and creation of individual patient profiles [56]. In this study, we promote the use of biomarkers to assess the potential use of specific drugs in stratified therapeutic groups.

## Competing interests

The authors declare that they have no competing interests.

## Authors’ contributions

SB carried out the cell culturing experiments and data analysis and contributed to the draft of the manuscript. CS carried out the HRM analysis, VP worked on the sensitivity of cell lines using rapamycin, AF participated in experimental design and critical review of the manuscript, NB did the work on the sensitivity of the BRCA1 mutant cell line and GG conceived the study, designed and coordinated the project and contributed to the writing of the manuscript. All authors read and approved the final manuscript.
